# Stress and displacement patterns in the craniofacial skeleton with rapid maxillary expansion—a finite element method study

**DOI:** 10.1186/s40510-017-0172-2

**Published:** 2017-07-10

**Authors:** J. Priyadarshini, C. M. Mahesh, B. S. Chandrashekar, Abhishek Sundara, A. V. Arun, Vinay P. Reddy

**Affiliations:** 1 Private Practice, Bellary, Karnataka India; 2 Department of Orthodontics and Dentofacial Orthopaedics, Krishnadevaraya College of Dental Sciences, Hunasamaranahalli, New Airport Road, Bangalore, 562157 Karnataka India

**Keywords:** Finite element analysis, Rapid Maxillary Expansion, Craniofacial sutures, Displacement

## Abstract

**Background:**

Rapid maxillary expansion (RME), indicated in the treatment of maxillary deficiency directs high forces to maxillary basal bone and to other adjacent skeletal bones. The aim of this study is to (i) evaluate stress distribution along craniofacial sutures and (ii) study the displacement of various craniofacial structures with rapid maxillary expansion therapy by using a Finite Element model.

**Methods:**

An analytical model was developed from a dried human skull of a 12 year old male. CT scan images of the skull were taken in axial direction parallel to the F-H plane at 1 mm interval, processed using Mimics software, required portion of the skull was exported into stereo-lithography model. ANSYS software was used to solve the mathematical equation. Contour plots of the displacement and stresses were obtained from the results of the analysis performed.

**Results:**

At Node 47005, maximum X-displacement was 5.073 mm corresponding to the incisal edge of the upper central incisor. At Node 3971, maximum negative Y-displacement was -0.86 mm which corresponds to the anterior zygomatic arch, indicating posterior movement of craniofacial complex. At Node 32324, maximum negative Z-displacement was -0.92 mm representing the anterior and deepest convex portion of the nasal septum; indicating downward displacement of structures medial to the area of force application.

**Conclusions:**

Pyramidal displacement of maxilla was evident. Apex of pyramid faced the nasal bone and base was located on the oral side. Posterosuperior part of nasal cavity moved minimally in lateral direction and width of nasal cavity at the floor of the nose increased, there was downward and forward movement of maxilla with a tendency toward posterior rotation. Maximum von Mises stresses were found along midpalatal, pterygomaxillary, nasomaxillary and frontomaxillary sutures.

## Background

Orthodontics and dentofacial orthopedics have changed from an opinion-based practice to evidence-based practice. Rapid maxillary expansion (RME) is indicated in the treatment of maxillary deficiency. During RME, high forces directed to the maxillary basal bone and perhaps to other adjacent skeletal bones can easily split the mid-palatal suture in young individuals and force the two maxillary halves laterally. Midfacial orthopedic expansion has been recommended for use in conjunction with protraction forces on the maxilla because it disrupts the circum-maxillary sutural system and presumably facilitates the orthopedic effect of the facemask [1, 2]. Various craniofacial areas especially in the areas of articulation of the maxilla are also effected [3]. A recent study used cone-beam computed tomography imaging to conclude that RME resulted in forward movement of the maxilla as well and also vertical dentoalveolar changes [4].

It is still an enigma as to where these areas of maximum force concentration are and how these heavy forces get dissipated. Sutures undergo anabolic changes such as increased sutural width, angiogenesis, and bone apposition in response to anteriorly directed forces. Studies provided histologic descriptions of sutural response to both tensile and compressive forces. Bone formation has been observed at the edges of the mid-palatal suture after rapid palatal expansion [5].

In recent years, finite element method (FEM) has been a powerful research tool for solving various structural mechanical problems. It is recognized as a general procedure for mechanical approximation to all physical problems that can be modeled by differential equation description [6].

Previous study assessed the stress and displacement in the maxilla alone [7]. The present study encompasses the changes produced in the entire craniofacial complex and was planned to explore how heavy transverse orthopedic forces generated by RME get dissipated within the craniofacial complex and to evaluate the pattern of stress accumulation, dissipation, and displacement of various vcraniofacial structures with RME appliance using a three-dimensional FEM study by using a model that better represents the human skull than previously presented.

### Aims and objectives

The aims of this study are as follows:

1. To evaluate stress distribution along craniofacial sutures
2. To study the displacement of various craniofacial structures with RME therapy by using a finite element model

## Methods

**Fig. 1:**
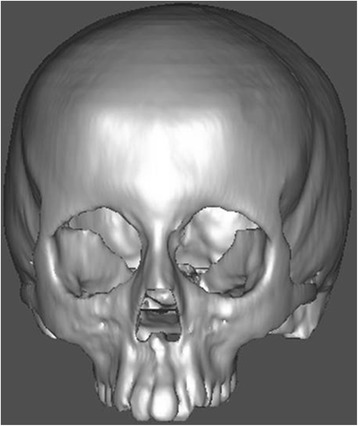
Dried young human skull of a 12-year-old male

Computed tomography (CT) scan images of a 12-year-old boy’s cranium were taken by Spiral CT Scan Machine (X-Force/SH spiral CT scan machine manufactured by Toshiba) (Fig. 1). The entire skull excluding the mandible were sliced at 1-mm interval, and nodes are assigned to the bones and sutures of interest. A 3D CAD model was created using Mimics software. Mimics software helps to import and visualize and helps in 3D rendering, 3D information, reslicing, and measuring the CT scan details.

**Fig. 2:**
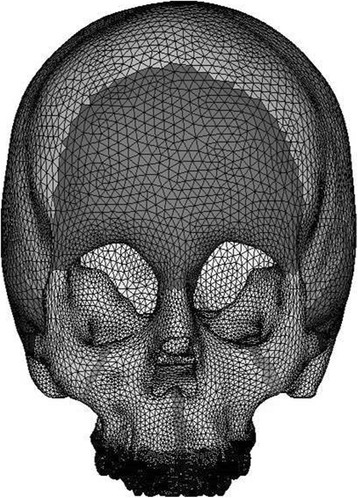
Frontal view of the three-dimensional FE model

**Fig. 3:**
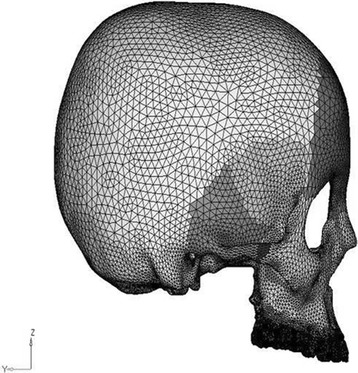
Lateral view of the three-dimensional FE model

**Fig. 4:**
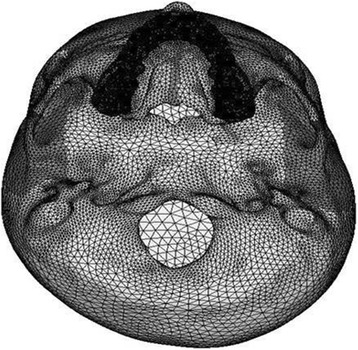
Basal view of the three-dimensional FE model

**Fig. 5:**
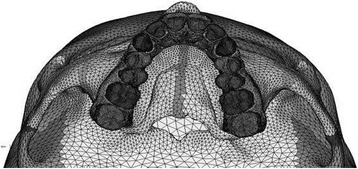
Palatal view of the three-dimensional FE model

A 3D CAD model was imported into Hypermesh Software. The model was discretized into finite number of elements and nodes as per the anatomy, and the craniofacial structures were modeled (sutures and bones). The analytical model is shown in Figs. 2, 3, 4, and 5.

CT scan images of the skull were taken in axial direction parallel to the F-H plane at 1-mm interval. CT scan images containing cloud data point information were processed using Mimics software, and the required portion of the skull was exported into a stereo-lithography model. Files in stereo-lithography (STL) format were imported into rapid form software to create the surface data. Then, the surface model in IGES (Initial Graphics Exchange Specification) format was exported to HYPERMESH. The process of converting a geometric model into a finite element model is called meshing. The finite element model consists of 115,694 nodes and 537,684 elements. In this study, the model consisted of 694,164 degree of freedom. The discretized FE model is shown in different views in Fig. 2 (frontal view of the three-dimensional FE model), Fig. 3 (lateral view of the three-dimensional FE model), Fig. 4 (basal view of the three-dimensional FE model), and Fig. 5 (palatal view of the three-dimensional FE model).

**Fig. 6:**
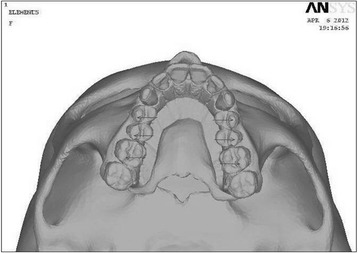
Force of 19 kg/mm^2^ was applied over the crown of premolars and first molar

The material properties (Young’s modulus and Poisson’s ratio) of the tooth, cortical bone, and PDL (periodontal ligament) were entered in the pre-processing stage. The assembled finite element model of the skull, tooth, and PDL was then imported into ANSYS software for analysis. A known force of 19 kg/mm^2^ was applied over the crown of premolars and first molar as shown in Fig. 6.

ANSYS software was used to solve the mathematical equation and to calculate the stress and displacement pattern of the skull. Post processing was the last stage of the FEM in which contour plots of the displacement and stresses was obtained from the results of the analysis performed.

## Results

The biomechanical changes were evaluated under the following headings:

1. Displacement of different bones of craniofacial complex
2. Stress distribution among different bones and sutures

### Displacement pattern

The results of displacement patterns of various structures are shown in Table 1.

**Table 1:** Computational result of the transversal (X), sagittal (Y), and vertical (Z) displacements of the various skeletal structures of the craniofacial complex following 5 mm of transverse expansion on application of 19 kg/mm^2^ force

Region	Selected node	*X* (mm)	*Y* (mm)	*Z* (mm)
Dentoalveolar	Incisal edge of 1 (47,005)	5.073	0.79	0.42
	Cusp tip 3 (44,665)	5.068	0.65	0.28
	Cusp tip 6 (35,146)	5.068	0.69	0.28
	Apical region of 1 (106,126)	4.281	0.93	−0.62
	Apical region of 3 (99,505)	3.878	0.43	0.28
	Apical region of 6 (112,557)	4.273	0.37	0.32
Palate	Anterior part of palate (17,631)	3.077	1.21	−0.81
	Posterior part (17,161)	2.09	1.12	−0.98
Maxilla	Point “A” (32,324)	3.55	0.86	−0.92
	ANS (16,022)	3.078	1.24	−0.72
	Tuberosity (12,721)	3.26	0.55	0.32
	Zygomatic buttress (11,611)	2.89	0.019	0.85
	Inferior orbital rim (10,586)	1.97	−0.21	0.90
	Frontal process (14,235)	1.00	0.078	−0.22
Nasal cavity wall	Antero-inferior (17,902)	2.95	0.43	0.02
	Anterosuperior (14,894)	1.41	0.21	−0.02
	Posteroinferior (14,131)	1.93	0.42	−0.35
	Posterosuperior (17,005)	0.49	0.009	−0.04
Nasal bone	Body (16,523)	0.31	−0.63	−0.71
Sphenoid bone	Lateral pterygoid inferior (24,419)	1.91	0.63	−0.52
	Lateral pterygoid superior (24,118)	0.651	0.06	0.18
	Medial pterygoid inferior (20,674)	1.88	−0.53	−0.39
	Medial pterygoid superior (24,011)	0.146	−0.09	0.23
	Greater wing (29,115)	0.33	−0.41	0.67
Zygomatic bone	Body (5320)	0.25	−0.83	1.64
	Frontal process (22,309)	2.19	−1.2	1.62
	Zygomatic arch anterior (3971)	0.55	−0.86	1.72
	Zygomatic arch posterior (3907)	0.045	−0.42	0.08
Frontal bone	Supraorbital (7846)	0.015	−0.02	0.12
	Forehead (18,937)	0.02	−0.03	0.065
Temporal bone	Squamous (3786)	0.55	−0.28	0.36
Parietal	Tuberosity (31,299)	0.09	−0.27	0.41
Occipital	Squamous (20,670)	0.014	−0.09	0.03

#### Displacement in the transverse plane (*X*-displacement)

Node 47,005 mm corresponding to the incisal edge of the upper central incisor showed maximum *X*-displacement (lateral displacement), and it was measured to be 5.073 mm. From the frontal view, the pyramidal displacement of the maxilla was evident. The apex of the pyramid faced the nasal bone, and the base was located on the oral side.

Viewed occlusally, the two halves of the maxillary dentoalveolar complex, basal maxilla, and lateral walls of the nasal cavity separated more widely, anteriorly. The posterosuperior part of the nasal cavity had moved minimally in the lateral direction, and the width of the nasal cavity at the floor of the nose increased markedly. There was no significant lateral displacement observed at the temporal, parietal, frontal, sphenoid, and occipital bones.

#### Displacement in the antero-posterior plane (*Y*-displacement)

Node 3971 which corresponds to the anterior zygomatic arch showed a maximum negative *Y*-displacement (backward displacement) of −0.86 mm indicating that this portion of the craniofacial complex has moved posteriorly.

At node 16,022, which represents the antero-inferior border of the nasal septum, a maximum positive *Y*-displacement (forward displacement) measuring 1.2402 mm was noted. Maxillary bone, maxillary central incisors, and molars were slightly displaced forward. A backward displacement occurred in relation to the zygomatic bone.

#### Displacement in the vertical plane (*Z*-displacement)

Node 32,324, representing the anterior deepest convex portion of the nasal septum maximum, showed a negative *Z*-displacement (downward displacement) of −0.92 mm, indicating a downward displacement of structures medial to the area of force application. At node 3971, corresponding to the anterior zygomatic arch, a maximum positive *Z*-displacement (upward displacement) of 1.72 mm was seen.

It suggests that the nasomaxillary complex rotated in such a manner that the lateral structures had moved upward and midline structures downward. The maxillary central incisors and the anterior part of the maxillary bone (ANS and point A) were displaced downward.

#### Stress distribution among different bones and sutures

The magnitude and distribution of von Mises stresses produced at various sutures of the craniofacial complex by the activation of the RME device up to 5 mm on each side are shown in Tables 2 and 3.

**Table 2:** Computational result of von Mises stress contours of the various skeletal structures

Bones	VM stress, max (kg/mm^2^)	VM stress, min (kg/mm^2^)	VM stress, average (kg/mm^2^)
Frontal bone	42.74	0.285	21.51
Maxillary bone	58.7	0.747	29.7
Nasal bone	51.7	3.628	27.66
Sphenoid bone	20.96	0.25	10.605
Temporal bone	26.905	0.15	13.52
Zygomatic bone	44.602	0.573	22.58

**Table 3:** Computational result of von Mises stress contours of the various sutural structures

Sutures	VM stress, max (MPa)	VM stress, min (MPa)	VM stress, average (kg/mm^2^)
Frontomaxillary	37.8	1.416	19.608
Frontonasal	15.978	2.849	9.41
Frontozygomatic	13.39	3.034	8.212
Internasal	41.602	3.984	22.79
Laminacribosa	7.107	0.966	4.036
Mid-palatal	59.532	1.531	30.53
Nasomaxillary	25.51	3.103	14.306
Pterygomaxillary	46.495	6.336	26.41
Spheno maxillary	39.738	2.663	21.2
Sphenozygomatic	19.575	1.045	10.31
Temporozygomatic	21.266	0.308	10.78
Zygomaticomaxillary	36.558	3.898	20.228

In the crown of the first permanent molar of maxillary region, a compressive stress of 58.7 kg/mm^2^ was observed. The rest of the dentoalveolar regions from canine to molar also experienced high initial stresses of 21.51–29.7 kg/mm^2^.

Areas of high stresses were seen in the region of maxillary bone and maxillary tuberosity. High stresses also were found around the zygomatic process of the maxilla, nasomaxillary suture, nasofrontal suture, and frontomaxillary suture. Compressive forces of up to 25.51 kg/mm^2^ were experienced in the nasal bone, nasomaxillary suture, and nasofrontal suture. The fields of high stresses were around the frontozygomatic suture and almost the whole length of the frontal process of the zygomatic bone. The anterior rim of the frontal process of the zygomatic bone received 37.8 kg/mm^2^ of forces. Similarly, the zygomatic arch and the area of the mid-palatal suture (59.532) experienced high levels of stress.

An interesting finding of this study was the presence of high stress all along the maxillary bone (58.7), radiating upward to deeper anatomic structures such as the body of the sphenoid bone. In the frontal, parietal, temporal, and occipital bones, RME produced stress levels ranging from 0.285 to 1.085 kg/mm^2^.

## Discussion

Skeletal Class III malocclusions appear in various conditions and patterns. It is caused by abnormal growth of the jaws or growth disharmony, as overdevelopment of the mandible, underdevelopment of the maxilla, or a combination of both. There are many controversies concerning the treatment modalities and treatment timing of skeletal Class III malocclusions with respect to skeletal and dental discrepancy, age, and residual growth. A reduction in the growth of the maxilla is caused not only by the antero-posterior divergence but also by a transverse variation, resulting, in many cases, in posterior crossbites [8].

Haas reported on the orthopedic effect of RME, which produced a forward and downward tipping of the maxilla with concomitant downward and backward mandibular rotation [9]. These orthopedic changes facilitated the correction of a mild Class III malocclusion. RME is effective for correction of transverse discrepancies and also for protraction of the maxilla by remodeling the nine circumaxillary sutures [6] .

When Yu et al. compared the effect of maxillary protraction with and without RME, they concluded that opening the mid-palatal suture using a RME appliance and directing the protraction force inferiorly from the occlusal plane, passing through the maxillary center of resistance and also through the apical portion of the first premolar, maxillary protraction that is similar to normal downward and forward growth of the maxilla can be effectively achieved [8].

In yet another study by Park et al. to analyze displacement and stress distribution in the maxilla during maxillary expansion followed by protraction using bone-borne and conventional tooth-borne palatal expanders and a facemask via three-dimensional finite element analysis, they concluded that the group with only facemask resulted in more anterior displacement of the maxilla than the combination of facemask and bone-borne expanders. Further, tooth-borne expanders reported more anterior movement of the maxilla when protraction was combined with expansion and compared with protraction without expansion. This is due to the synergistic effect when combined with the facemask as reported by Yu et al. [8].

The three-dimensional FEM used in the present study provides the freedom to simulate orthodontic force systems applied clinically and allows analysis of the response of the craniofacial skeleton to the orthodontic loads in three-dimensional space. The FEM has several advantages, and the study can be repeated as many times as the operator wishes [10].

Sequential CT scan images were taken at 1-mm intervals to reproduce finer and detailed aspects of the geometry. Previous studies reported sections at 10-mm [11, 12], 5-mm [13, 14], and 2.5-mm [15] intervals. This study differs from previous studies in the type of element used for meshing and solid tetrahedral elements used, each having 10 nodes, giving better stress transmissibility and bending deformations. Therefore, this model is a better representation of a real human skull than the previous models [13, 14].

### Displacement of various craniofacial structures with rapid maxillary expansion by using finite element analysis

Maximum lateral displacement was at the incisal edge of the upper central incisor. The pyramidal displacement of maxilla was evident. The apex of the pyramid faced the nasal bone, and the base was located on the oral side. The posterosuperior part of the nasal cavity had moved minimally in the lateral direction, and the width of the nasal cavity at the floor of the nose increased markedly, whereas no significant lateral displacement was observed at the temporal, parietal, frontal, sphenoid, and occipital bones. The studies done by Haas et al., Jafari et al. [13], Cleall et al. [16], Davis and Kronman [17], and Iseri et al. [14] showed similar findings.

The maximum negative *Y*-displacement (backward displacement) was observed at the anterior zygomatic arch, indicating that this portion of the craniofacial complex had moved posteriorly. In a similar study done by Jafari et al. [13], the maximum negative *Y*-displacement was less compared to our study. The difference may be due to (i) CT scan images were taken at 5-mm interval, (ii) analysis of the complete skull was not considered as it was thought that the analysis of one half of the cranium will mirror similar results on the opposite side, and (iii) a known force was not applied.

The present study suggests that the resistance of the mid-palatal opening is probably not in the suture itself but in the surrounding structures of the sphenoid and zygomatic bones, thus confirming the studies done in the past by Isaacson and Ingram [18] and Wertz [19].

When the mid-palatal suture opens with RME therapy, the maxilla always moves downward and forward. This is probably due to the disposition of the maxilla-cranial sutures [1]. This was demonstrated by anterior and downward displacement of point A, ANS, and prosthion. This is in accordance with previous studies [13, 14, 19, 20].

The pterygoid plates can bend only to a limited extent with pressure, and this confining effect of the pterygoid plates of the sphenoid minimizes the ability of the palatine bones to separate at the midsagittal plane [18].

An increase in nasal width has been demonstrated as a response to RME [1, 2, 19, 21]. The numerical results demonstrate that the width of the nasal cavity at the floor of the nose increased markedly compared to the superior part. This result is similar to Pavlin and Vukicevik’s study [22]. Therefore, a combination of increase in nasal width, lowering of palatal plane, and probably straightening of the nasal septum after RME can help the patients with nasal stenosis, by increasing air flow.

This study also provided additional explanation about the bony tissue mechanical reactions, which are the first steps in the compound process of tissue response to jaw expansion.

### Evaluation of stress distribution along craniofacial sutures

The von Mises stresses were used for this analysis because of the appropriateness and the validity of the von Mises theory of failure [14].

The present study showed that the mid-palatal suture had experienced high levels of stress (59.532 MPa). An interesting finding of this study was the presence of high stress all along the maxillary bone (58.7 MPa), radiating upward to deeper anatomic structures such as the body of the sphenoid bone.

Mechanical stresses elicited by exogenous forces are experienced and transmitted by sutures [23]. Sutural strain patterns are similar between dry skull models and the same structures in vivo [23]. Sutures have a range of mobility and certainly are not immovable joints when they are patent. Mechanical stresses experienced in sutures, with the right characteristics, can modulate sutural growth [24]. This had been proved time and again. Our present study also confirmed this.

The present study concludes that mid-palatal suture shows a maximum von Mises stress followed by pterygomaxillary, nasomaxillary, and frontomaxillary sutures in the descending order of frequency. This is contrary to studies done by Jafari et al. [13]. The difference observed may be attributed to the sample used for the generation of FE model and selection of nodes and elements. In the present study, a known force is applied instead of known displacement.

The study done by Shetty et al. [25] showed that in the anterior region, the forces spread superomedially along the frontal process of the maxilla and the medial orbital wall up to the junction of the nasal and lacrimal bones. The primary stresses in their study radiated laterally to the zygomaticomaxillary and the zygomaticofrontal sutures, which is contrary to our findings. This difference may be attributed to discretization process during FE model generation and selection of nodes and elements.

In this study, the lateral stresses mainly radiated to the zygomaticotemporal and the sphenozygomatic sutures and it has been demonstrated that the pattern of stress distribution was different along the various craniofacial sutures in response to RME. Both tensile and compressive stresses of variable magnitude were demonstrated along the same suture. The zygomaticomaxillary, zygomaticotemporal, and zygomaticofrontal sutures were associated with both tensile and compressive stresses. Sutural growth is accelerated by both tension and compression with appropriate parameters such as strain amplitude, rate, and dose [23].

The presence of differential strain patterns suggests the possibility of differential bone remodeling along the same suture. Differential strain patterns and magnitudes along the same suture were documented by Oberheim and Mao [26] who showed contrasting bone-strain patterns in zygomatic arch across zygomaticotemporal suture—i.e., tensile on its lateral surface and compressive on its medial surface—suggesting potentially differential growth responses of zygomatic arch with headgear therapy.

The absolute level of induced stresses greatly depends on bone elasticity and patient’s age. With the same orthopedic load, equivalent sutures of juvenile skulls experience significantly higher bone strain than adult skulls, suggesting that same mechanical force might have different biologic effects on immature and mature facial skeletons.

It may be noted that any variation in values between this study and other studies may be attributed to the sample used for the generation of FEM or the model generated on the computer (or both) or selection of the nodes and elements on the FEM (or both).

## Conclusions

The conclusions are as follows:

1. Pyramidal displacement of the maxilla was evident. The apex of the pyramid faced the nasal bone, and the base was located on the oral side.
2. Posterosuperior part of the nasal cavity moved minimally in the lateral direction, and width of the nasal cavity at the floor of the nose increased markedly.
3. There were downward and forward movements of the maxilla with a tendency toward posterior rotation.
4. Pterygoid plates were displaced laterally.
5. In the frontal plane, the center of rotation of the maxilla was approximately near the superior orbital fissure.
6. Maximum von Mises stresses were found along mid-palatal, pterygomaxillary, nasomaxillary, and frontomaxillary sutures in a descending order of frequency.
7. Zygomaticomaxillary, zygomaticotemporal, and zygomaticofrontal sutures were associated with both tensile and compressive stresses.
8. Bilateral pterygomaxillary and zygomatic buttress osteotomies are essential when carrying out surgically assisted RME in adults.
9. The mid-palatal suture had experienced high levels of stress; an interesting finding of this study was the presence of high stress all along the maxillary bone, radiating upward to deeper anatomic structures such as the body of the sphenoid bone.

## Abbreviations

CAD: Computer-assisted design
CT: Computed tomography
FE: Finite element
FEM: Finite element method
IGES: Initial Graphic Exchange Specification
PDL: Periodontal ligament
RME: Rapid maxillary expansion
STL: Stereo-lithography

## References

[1] Haas AJ (1970). Palatal expansion: just the beginning of dentofacial orthopaedics. Am J Orthod.

[2] Haas AJ (1965). Treatment of maxillary deficiency by opening the mid-palatal suture. Angle Orthod.

[3] Zimring JF, Isaacson RJ (1965). Forces produced by rapid maxillary expansion. Part II. Forces present during retention. Angle Orthod.

[4] Habeeb M, Boucher N, Chung CH (2013). Effects of rapid palatal expansion on the sagittal and vertical dimensions of the maxilla: a study on cephalograms derived from cone-beam computed tomography. Am J Orthod Dentofacial Orthop.

[5] Brandt HC, Shapiro PA, Kokich VG (1979). Experimental and post experimental effects of posteriorly directed extraoral traction in adult Macaca fascicularis. Am J Orthod.

[6] Korioth TWP, Versluis A (1997). Modelling the mechanical behaviour of the jaws and their related structures by finite element (FE) analysis. Crit Rev Oral Biol Med.

[7] Dalband M, Kashani J, Hashemzeh H. Three-dimensional finite element analysis of stress distribution and displacement of the maxilla following surgically assisted rapid maxillary expansion with tooth- and bone-borne devices. Journal of Dentistry, Tehran University of Medical Sciences, Tehran, Iran. 2015;12(4):298–306. PMC4662768 26622285

[8] Yu HS, Baik HS, Sung SJ, Kim KD, Cho YS (2007). Three-dimensional finite-element analysis of maxillary protraction with and without rapid palatal expansion. Eur J Orthod.

[9] Haas AJ (1961). Rapid expansion of the maxillary dental arch and nasal cavity by opening the midpalatal suture. Angle Orthod.

[10] Park J, Bayome M, Zahrowski JJ, Kook YA (2017). Displacement and stress distribution by different bone-borne palatal expanders with facemask: a 3-dimensional finite element analysis. Am J Orthod Dentofacial Orthop.

[11] Tanne K, Sakuda M (1991). Biomechanical and clinical changes of the craniofacial complex from orthopedic maxillary protraction. Angle Orthod.

[12] Tanne K, Hiraga J, Sakuda M (1989). Effects of directions of maxillary protraction forces on biomechanical changes in craniofacial complex. Eur J Orthod.

[13] Alireza Jafari K, Sadashiva S, Mohan K (2003). Study of stress distribution and displacement of various craniofacial structures following application of transverse orthopedic forces—a three-dimensional FEM study. Angle Orthod.

[14] Iseri H, Tekkaya AE, Oztan O, Bilgiç S (1998). Biomechanical effects of rapid maxillary expansion on the craniofacial skeleton, studied by the finite element method. Eur J Orthod.

[15] Pawan G, Ashima V, Raviraj A (2007). Stress and displacement patterns in the craniofacial skeleton with rapid maxillary expansion: a finite element method study. Am J Orthod Dentofacial Orthop.

[16] Cleall JF, Bayne D, Posen J, Subtelny JD (1965). Expansion of the mid-palatal suture in the monkey. Angle Orthod.

[17] Davis WM, Kronman JH (1969). Anatomical changes induced by splitting of the mid-palatal suture. Angle Orthod.

[18] Isaacson RJ, Ingram AH (1964). Forces produced by rapid maxillary expansion. Angle Orthod.

[19] Wertz RA (1970). Skeletal and dental changes accompanying rapid mid-palatal suture opening. Am J Orthod.

[20] Gardner GE, Kronman JH (1971). Cranioskeletal displacement caused by rapid palatal expansion in the rhesus monkey. Am J Orthod.

[21] Memikoglu TUT, Iseri H (1999). Effects of a bonded rapid maxillary expansion appliance during orthodontic treatment. Angle Orthod.

[22] Pavlin D, Vukicevic D (1984). Mechanical reactions of facial skeleton to maxillary expansion determined by laser holography. Am J Orthod.

[23] Mao JJ, Wang X, Kopher RA (2003). Biomechanics of craniofacial sutures: orthopedic implications. Angle Orthod.

[24] Mao JJ (2002). Mechanobiology of craniofacial sutures. J Dent Res.

[25] Shetty V, Caridad JM, Caputo AA, Chaconas SJ (1994). Biomechanical rationale of surgical orthodontic expansion of the adult maxilla. J Oral Maxillofacial Surg.

[26] Oberheim MC, Mao JJ (2002). Bone strain pattern of the zygomatic complex in response to simulated orthopedic forces. J Dent Res.

